# Development of a Nomogram for Predicting Depression in the Elderly Using Patient Health Questionnaire-9 among a Nationwide Sample of Korean Elderly

**DOI:** 10.3390/jpm11070645

**Published:** 2021-07-07

**Authors:** Haewon Byeon

**Affiliations:** Department of Medical Big Data, College of AI Convergence, Inje University, Gimhae-si 50834, Gyeonsangnam-do, Korea; bhwpuma@naver.com; Tel.: +82-10-7404-6969

**Keywords:** depression, nomogram, patient health questionnaire, multiple risk factors, epidemiological survey, high-risk group

## Abstract

This cross-sectional study developed a nomogram that could allow medical professionals in the primary care setting to easily and visually confirm high-risk groups of depression. This study analyzed 4011 elderly people (≥60 years old) who completed a health survey, blood pressure, physical measurement, blood test, and a standardized depression screening test. A major depressive disorder was measured using the Korean version of the Patient Health Questionnaire (PHQ-9). This study built a model for predicting major depressive disorders using logistic regression analysis to understand the relationship of each variable with major depressive disorders. In the result, the prevalence of depression measured by PHQ-9 was 6.8%. The results of multiple logistic regression analysis revealed that the major depressive disorder of the elderly living alone was significantly (*p* < 0.05) related to monthly mean household income, the mean frequency of having breakfast per week for the past year, moderate-intensity physical activity, subjective level of stress awareness, and subjective health status. The results of this study implied that it would be necessary to continuously monitor these complex risk factors such as household income, skipping breakfast, moderate-intensity physical activity, subjective stress, and subjective health status to prevent depression among older adults living in the community.

## 1. Introduction

The prevalence of geriatric depression has been increasing due to the aging population [1]. The World Health Organization (2017) [2] forecasted that the prevalence of geriatric depression (60 years or older) worldwide has risen from 12% in 2015 to at least 22% in 2050, an almost two-fold increase. In particular, as the prevalence of depression in older adults has greatly increased while the global population is rapidly aging, depression has become a serious health problem for older adults [3]. The WHO [4] reported that the prevalence of depression among the global elderly population (≥60 years) was 12% in 2015. If this trend continues, it is predicted that one out of four older adults people (21.2%) would suffer from depression in 2050 [4]. In particular, it has been reported that the prevalence of major depressive disorders among adults in South Korea is lower than that in the United States and that in Europe, but the prevalence of the major depressive disorders among older adults in South Korea was relatively higher [5]. In a national survey of the elderly 65 years and older in South Korea [6,7], the prevalence of depressive disorders was 33.1–34.8%. Since South Korea is rapidly becoming an aging society, paying attention to the problems of older adults, who account for a majority of depressive disorder occurrences, is unavoidable.

Since the prevalence of depressive disorders is high and they cause various functional disorders, it is important to detect them early. Depressive disorders increase physical diseases and mortality because they worsen the performance of social functions and quality of life and adversely affect physical and mental health [8]. In addition, since the onset of depressive symptoms is greatly affected by socio-cultural factors and health behaviors, it is necessary to prepare mental health policies optimized for each country. It is critical to conduct studies for predicting depressive disorders using reliable epidemiological data that can represent the population. Nevertheless, it is hard to differentiate depressive symptoms found in older adults from the symptoms due to aging such as a decrease in hormones, so it is not easy to detect high-risk groups of geriatric depression at an early stage and continuously manage them in the community.

Previous studies have reported that gender, age, low education level, social and economic status, undesirable lifestyle habits (e.g., smoking and excessive drinking), marital status, chronic diseases, and psychological stress affect geriatric depression [9,10,11]. However, the limitations of these previous studies are that (1) they were conducted in a single area or a small group of older adults [11] and (2) most were limited to exploring individual risk factors for depression in older adults [9,10]. Recent studies revealed that health risk behaviors tended to group together [12,13] and 17.6% of Brazilian men [14], 23% of UK men [15], and 15.2% of South Korean men [16] were exposed to at least three health risk behaviors, such as drinking, smoking, and obesity, at the same time. Therefore, the consideration of multiple health risk behaviors is required when developing a model for predicting major depressive disorders in order to present practical data that are necessary to detect high-risk groups at an early stage and prevent these disorders, based on evidence from older adults living in a community.

As far as we know, no study has developed a nomogram for predicting high-risk groups of geriatric depression while considering multiple health risk behaviors, using epidemiological data that can represent older adults living in local communities in South Korea. This study identified risk factors that could influence geriatric disorders among various aspects including physical activities and nutritional factors, preventative factors, sociodemographic factors and depression risk factors (e.g., health risk behaviors) confirmed in previous studies [17,18,19] by using the Patient Health Questionnaire (PHQ-9) [20,21], a standardized depression screening test widely used for epidemiological surveys globally. This study developed a nomogram that could allow medical professionals in the primary care setting to easily and visually confirm high-risk groups of depression.

## 2. Materials and Methods

### 2.1. Data Source

This cross-sectional study is a secondary data analysis study using raw data from the 7th National Health and Nutrition Examination Survey conducted from 2016 to 2018, supervised by the Korea Centers for Disease Control and Prevention under the Ministry of Health and Welfare. The National Health and Nutrition Examination Survey is a set of national statistics supervised by the Ministry of Health and Welfare and the Korea Centers for Disease Control and Prevention and is government-designated (approval number 1702) based on Article 17 of the Statistics Act. It was conducted after receiving written consent from participants and with the approval of the Institutional Bioethics Committee of the Korea Centers for Disease Control and Prevention (No.1041107-201806-HR-011-01). This study used the population living in South Korea and selected survey targets by using the stratified cluster sampling method and the systematic sampling method, based on the 2010 Population and Housing Census data (complete enumeration). The Seventh National Health and Nutrition Examination Survey investigated 24,269 people from 13,248 households in 576 surveyed districts, and the participation rate was 76.7% (*n* = 18,614). The National Health and Nutrition Examination Survey examines disease morbidity, activity restriction, quality of life, health behavior, and physical activity, and was conducted by interviews and a self-recording method during the survey period. A nutritional survey was performed by having a nutrition surveyor visit the home of the subject in person and conducting a food intake frequency survey using the interview method. This study analyzed 4011 older adults people (≥60 years old) who completed the health survey, blood pressure, physical measurement and blood tests, and a PHQ-9 (standardized depression screening test) [21].

### 2.2. Measurement and Definition of Variables

The dependent variable of this study was the prevalence of a major depressive disorder, measured using the Korean version of PHQ-9 [21]. PHQ-9 is a standardized depression screening test developed by Spitzer et al. (1999) [20] to diagnose mental health in primary health care centers. It is composed of nine items corresponding to the diagnostic criteria of the Diagnostic and Statistical Manual of Mental Disorders (DSM-IV) for major depressive disorders. The PHQ-9 is a self-report test, and has excellent sensitivity and specificity [22]. Moreover, since it can simply check the severity of a major depressive disorder using only nine items, it has the advantage that it is highly likely to be applied to actual screening in epidemiological investigations as well as in the medical field [22]. The PHQ-9 asks a subject how often he or she has experienced anhedonia, depression, changes in sleep, fatigue, changes in appetite, guilt or worthlessness, decreased concentration, akathisia or feeling down, and suicidal thoughts in the past two weeks. It is evaluated on a four-point scale: “never”, “for a few days”, “more than one week”, and “almost every day”. The total score ranges from 0 to 27, and a higher score means more severe depression. The threshold of depression was defined as ten points (depression ≥10 points out of 27 points) based on the results of previous studies [23,24]. Choi (2017) [25] reported that the sensitivity and specificity of PHQ-9 were 81.1% and 89.9%, respectively. The reliability of the tool (Cronbach’s α) was 0.89.

The explanatory variables included sociodemographic characteristics, physical characteristics, nutritional characteristics, health behaviors, and health status, referring to previous studies [9,10,11,17,18,19]. Sociodemographic characteristics were gender (male/female), age (60–64, 65–69, 70–74, 75–79, or over 80), living with a spouse (yes or no), education level (“elementary school graduation or below”, “middle school graduation”, “high school graduation”, or “college graduation or above”), monthly mean household income (<KRW 1.5 million, ≥KRW 1.5 million and <KRW 2 million, ≥KRW 2 million and <KRW 3 million, or ≥KRW 3 million), and receiving national basic livelihood security (yes or no). Physical characteristics were waist circumference (cm), obesity by body mass index (BMI, kg/m^2^) (underweight (<18.5 kg/m^2^), normal (≥18.5 kg/m^2^ and < 23 kg/m^2^), pre-obesity stage (≥23 kg/m^2^ and <25 kg/m^2^), stage 1 obesity (≥25 kg/m^2^ and <30 kg/m^2^), stage 2 obesity (≥30 kg/m^2^ and <35 kg/m^2^), or stage 3 obesity (≥35 kg/m^2^)), and subjective body type perception (very thin, slightly skinny, average, slightly obese, or very obese). Nutritional characteristics were the mean frequency of having breakfast per week for the past year (rarely, “1–2 times per week”, “3–4 times per week”, or “5–7 times per week”), daily n-3 fatty acid intake (g/day), daily n-6 fatty acid intake (g/day), and daily vitamin c intake (mg/day). This study measured the n-3 fatty acid intake, n-6 fatty acid intake, and vitamin c intake by using the 24-h dietary recall method, and the survey data of food intake over one day were converted into a continuous variable and analyzed.

Health status variables were the usual level of stress awareness (“I hardly feel stressed”, “I feel stressed a little”, “I feel stressed a lot”, or “I feel stressed very much”), subjective health status (“bad”, “okay”, or “good”), hypertension (“normal”, “prehypertension”, or “hypertension”), diabetes (“normal”, “impaired fasting glucose”, or “diabetes”), hypercholesterolemia (no or yes), and hypertriglyceridemia (no or yes). Hypertension was defined as systolic blood pressure ≥140 mmHg or diastolic blood pressure ≥90 mmHg based on the mean value of the second and third measurements among three measurements by medical personnel using a sphygmomanometer. Among those not diagnosed with hypertension, prehypertension was defined as 120 mmHg ≤ systolic blood pressure < 140 mmHg and 80 mmHg ≤ diastolic blood pressure < 90 mmHg. Hypercholesterolemia was defined as a person currently taking a cholesterol-lowering drug or a person who had a total cholesterol level of 240 mg/dL or more measured while fasting for more than 8 h. Diabetes was defined as a person receiving a hypoglycemic agent/insulin injection after being diagnosed with diabetes by a doctor, or a person with a fasting blood sugar of 126 mg/dL or higher while fasting for 8 h or more. Impaired fasting glucose was defined as a person with a fasting blood sugar equal to or higher than 100 mg/dL and less than 126 mg/dL. Hypertriglyceridemia was defined as a person with a triglyceride content≥ 200 mg/dL via a blood test while fasting for 12 h or more. Health behaviors were drinking experience (yes or no), intemperance frequency (non-drinkers, once a month or fewer, once a week or fewer, or almost every day), smoking experience (current smokers, ex-smoker or non-smokers), control weight over the past year (“never tried to control weight”, “try to lose weight”, “try to maintain weight”, “try to gain weight”), moderate-intensity physical activity (yes or no), usual hours of sitting per day, usual minutes of sleep per day, and days of walking at least 30 min per week week (“never”, “1 day”, “2 days”, “3 days”, “4 days”, “5 days” “6 days”, or “7 days”) using a questionnaire. Physical activity was measured with the Korean version of GPAQ, a standardized Korean version of the Global Physical Activity Questionnaire (GPAQ) developed by the WHO [26].

### 2.3. Development of Depression Prediction Model

This study built a model for predicting major depressive disorders using logistic regression analysis to understand the relationship (influence) of each variable with major depressive disorders. The variable selection was made using the backward selection method, and this study presented the OR and 95%CI of an unadjusted model that did not adjust confounding factors, and those of an adjusted model that adjusted confounding factors.

**Figure 1:**
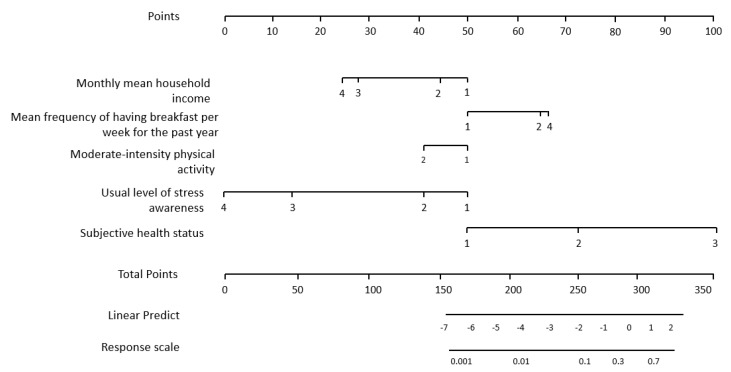
The nomogram for predicting depression in older adults in the community based on multiple risk factors.

This study developed a nomogram based on the developed depression prediction model (final model) so that clinicians could easily interpret the prediction result (prediction probability). The nomogram developed in this study consisted of four elements (Figure 1). First, a point line was presented. The point line is a line placed at the top of the nomogram to indicate a score falling in a risk class. In the case of a logistic nomogram, it consists of 0–100 points.

Second, a risk factor line was presented. This line indicates the range of a risk factor that affects the occurrence of an event. The number of risk factor lines is equal to the number of risk factors. Third, this study presents a probability line. The probability line is the sum of finally calculated nomogram scores, and it is placed at the bottom of the nomogram to derive the occurrence probability of a major depressive disorder. The fourth is the total point line, calculated and constructed based on a statistical model.

Monthly mean household income: 1 ≤ KRW 1.5 million, 2 ≥ KRW 1.5 million and < KRW 2 million, 3 ≥ KRW 2 million and < KRW 3 million, 4 ≥ KRW 3 million; Mean frequency of having breakfast per week for the past year: 1 = 5–7 times per week, 2 = 3–4 times per week, Rarely, 3 = 1–2 times per week, 4 = Rarely; Moderate-intensity physical activity: 1 = no, 2 = yes; Usual level of stress awareness: 1 = I feel stressed very much, 2 = I feel stressed a lot, 3 = I feel stressed a little,4 = I hardly feel stressed; Subjective health status:1 = good, 2 = normal, 3 = bad

### 2.4. Testing the Accuracy of a Nomogram for Predicting Geriatric Depression

Since the sample size of this model was not big enough (*n* = 4011) to validate the prediction model, this study used 10-fold cross-validation as a way to test the accuracy of the developed geriatric depression prediction nomogram to minimize the risk of overfitting, and presented the area under the curve (AUC), general accuracy, and calibration plot of each model. AUC is that of the receiver operating characteristic (ROC) curve. It is the most commonly used evaluation method in binary classification and is defined via diagnostic accuracy. A value closer to 1 means better diagnostic performance. The calibration plot is a figure for visually confirming the degree of agreement between the predicted probability in the nomogram and the observed probability.

## 3. Results

### 3.1. General Characteristics of Older Adults in the South Korean Community

The general characteristics of the 4011 subjects (56.4% were women and 43.6% were men) are presented in Table 1. Many subjects were non-drinkers (57.4%), non-smokers (61.3%), living with a spouse (71.5%), elementary school graduation or below (50.8%), a mean monthly household income less than KRW 1.5 million (42.8%), normal weight (46.3%), without moderate-intensity physical activity (96.4%), hypertension (58.8%), without diabetes (45.5%), without hypercholesterolemia (65.1%), and without hypertriglyceridemia (84.5%). The subjects usually sat 10.7 h per day on average (standard deviation 16.1), and slept 444.9 min per day on average (standard deviation 409.7). The prevalence of depression measured by PHQ-9 was 6.8%. Since only one person (0.1%) was in the stage 3 obesity class, it was merged with “stage 2 obesity” to make “stage 2 obesity or above” and the data were reanalyzed using chi-square and regression analyses.

### 3.2. Characteristics of Subjects According to the Prevalence of Depression

The characteristics of subjects according to the prevalence of depression are presented in Table 2. The results of the chi-square test revealed that the prevalence of a major depressive disorder was significantly (*p* < 0.05) affected by gender, current smoking status, marital status, education level, monthly mean household income, whether or not national basic livelihood security was received, subjective body type perception, the mean frequency of having breakfast per week for the past year, moderate-intensity physical activity, days of walking at least 30 min per week, the usual level of stress awareness, subjective health status, diabetes, hypertriglyceridemia, n-3 fatty acid intake (g), n-6 fatty acid intake (g), and vitamin C intake (mg).

### 3.3. Development of a Model for Predicting Geriatric Depression in the Community

The final model for predicting geriatric depression in the community is presented in Table 3. The results of univariate logistic regression analysis (unadjusted model) revealed that the major depressive disorder of older adults living alone was significantly (*p* < 0.05) related with monthly mean household income, the mean frequency of having breakfast per week for the past year, moderate-intensity physical activity, the subjective level of stress awareness, and subjective health status. The analysis results of the adjusted model confirmed both risk factors and protective factors of major depressive disorders (*p* < 0.05). The monthly mean household income was a protective factor for depression. Older adults with KRW 2–2.99 million had 32% less risk of depression (OR = 0.68, 95% CI: 0.40–1.14) than older adults with less than KRW 1.5 million, and older adults with KRW 3 million or more had 72% less risk of depression (OR = 0.28, 95% CI: 0.18 to 0.43) than older adults with less than KRW 1.5 million (*p* < 0.05). It was also confirmed that independent risk factors for depression were rarely having breakfast per week for the past year (OR = 2.14, 95% CI: 1.16, 3.95), no moderate-intensity physical activity (OR = 2.05, 95% CI = 1.15, 3.64), very high subjective stress OR = 14.17, 95% CI = 7.71, 26.02), a lot of subjective stress (OR = 8.00, 95% CI = 4.75, 13.46), a little subjective stress (OR = 2.18, 95% CI = 1.30, 3.66), okay subjective health status (OR = 2.65, 95% CI = 1.18, 5.95), and bad subjective health status (OR = 11.21, 95% CI = 5.14, 24.40) (*p* < 0.05).

### 3.4. Development and Validation of a Nomogram for Predicting Depression of Older Adults in the Community

**Figure 2:**
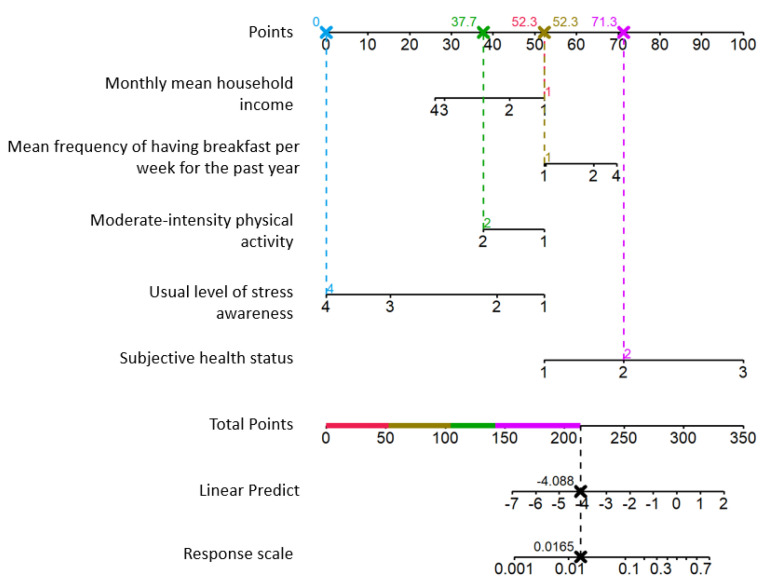
Application Example of Depression Prediction Nomogram for Older adults Living Alone: older adults who responded that mean monthly household income was less than KRW 1.5 million, mean frequency of having breakfast per week for the past year was 5–7 times a week, did not do moderate-intensity physical activity, and hardly felt stressed.

The nomogram for predicting depression in older adults in the community based on multiple risk factors is presented in Figure 1. Subjective stress awareness had the greatest influence among the risk factors for depression for older adults in the community. The older adults who responded that they felt stressed very much had the highest risk of a major depressive disorder. For example, in this depression prediction nomogram (Figure 2), it was predicted that the depression risk probability of older adults who responded that their mean monthly household income was less than KRW 1.5 million, mean frequency of having breakfast per week for the past year was 5–7 times a week, did not do moderate-intensity physical activity, and hardly felt stressed, was 1.6%.

**Figure 3:**
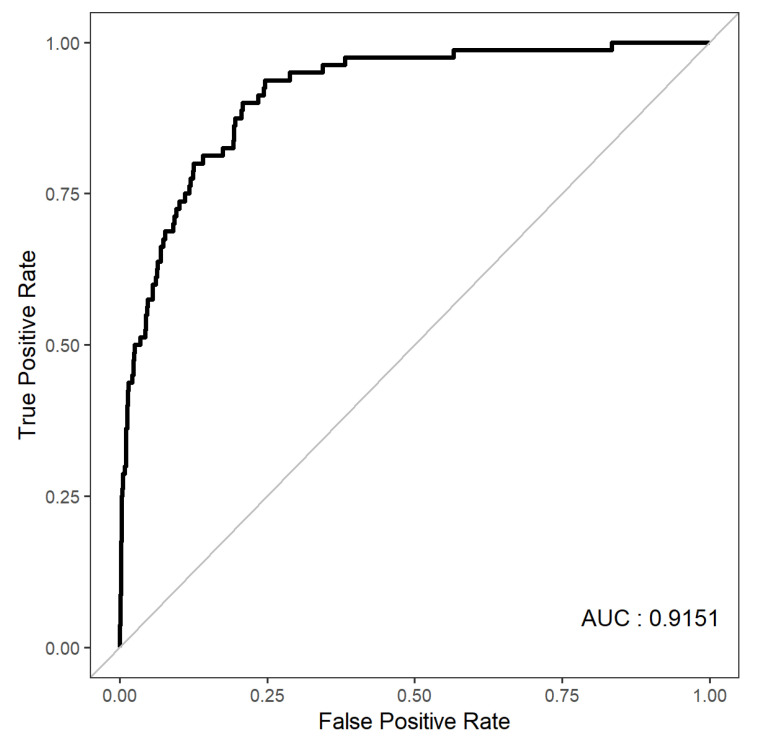
The AUC of the developed nomogram for predicting depression in older adults.AUC:the area under the curve.

**Figure 4:**
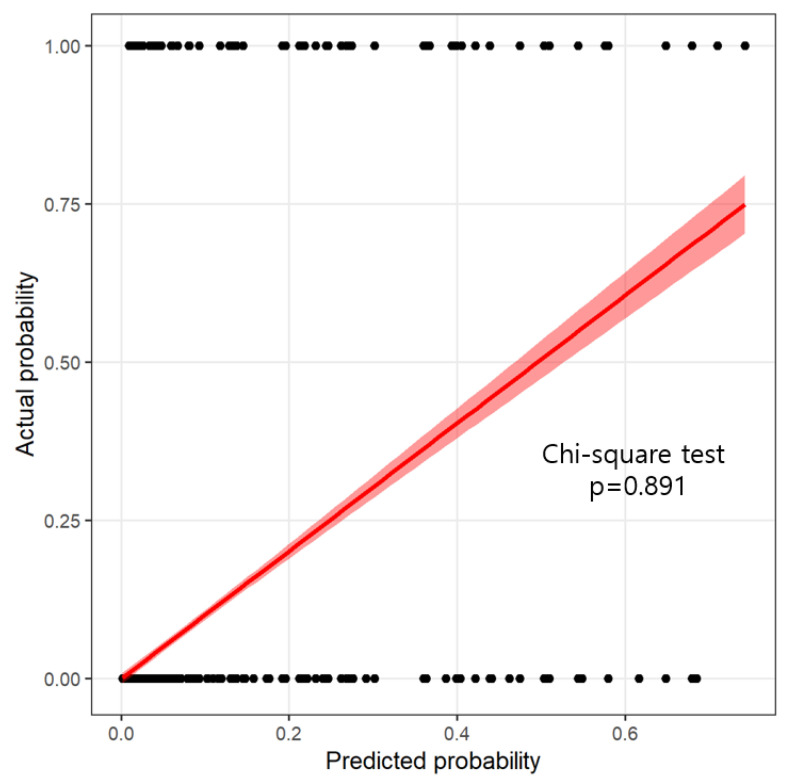
Calibration plot for test data: *X*-axis represents the predicted probability of major depressive disorder; *Y*-axis represents the actual major depressive disorder. An ideal (perfectly accurate) nomogram model would result in a plot in which the observed and predicted probabilities for given groups fall along the 45-degree line.

The developed nomogram for predicting depression of older adults was validated by using AUC, accuracy, and calibration plots. The AUC of the developed nomogram for predicting depression in older adults is presented in Figure 3. The results of 10-fold cross validation showed that the AUC and general accuracy of the nomogram were 0.91 and 0.96, respectively. This study compared predicted probability and observed probability using the calibration plot and the chi-square test for the group with depression and the group without depression (Figure 4) to find that there was no significant difference between predicted probability and observed probability (*p* = 0.891).

## 4. Discussion

This epidemiological study identified factors related to major depressive disorders in older adults in the community using PHQ-9. The results showed that a mean monthly household income of KRW 1.3 million won or more was an independent protective factor for depression. Skipping breakfast, absence of moderate-intensity physical activity, subjective stress, and subjective health status were independent risk factors for depression. It has been generally reported that a lower frequency of physical activity [27], lower socioeconomic status [28,29], higher stress [30], and poorer subjective health status [31] increase the prevalence of depression in the older adult population. Numerous previous studies [23,28,32] have suggested that regular physical activity was a major health promotion habit that can be critical in the prevention and treatment of depression. These studies have shown that physical activity can reduce the depression of individuals with diabetes, chronic stroke, or cancer as well as depression in healthy adults [28], that physically active older adults have less depression than physically inactive older adults [23], and that physical activity is effective in treating depression, preventing physiological side effects, and reducing the use of antidepressants [32].

Similar to the results of this study, Kim (2020) [33] evaluated 1447 older adults in South Korea and reported that the depression risk for older adults with a mean monthly household income of KRW 1.99 million or less was 5.4 times higher than that for older adults with a mean monthly income of KRW 4 million or more. The result agreed with the result of this study showing that a mean monthly household income of KRW 1.3 million or more was an independent protective factor against depression (older adults with KRW 1.3 million or more had a lower risk of depression than older adults with KRW 1.3 million or less). Regarding the relationship between income level and depression, Kang et al. (2008) [34] explained that income level had an overall effect on health status, including depression, because a low income level affected access to health care services, as an index reflecting an individual’s material status or resources to cope with a crisis.

Previous studies [23,28,32,33] that explored the risk factors of depression mainly identified individual risk factors for depression in the senile stage using regression analysis. Therefore, they were limited in understanding the multiple risk factors. This epidemiological study developed a nomogram to identify the multiple risk factors of depression in older adults living alone and predicted that older adults with an income level of KRW 1.3 million or less, skipping breakfast every day, no moderate-intensity physical activity, subjective perception of a lot of stress, and poor subjective health status had 85% depression risk, a very high risk. Therefore, detecting depression in this high risk group is required, among those who show these multiple risk factors at the same time, and continuous monitoring of this group is needed. Furthermore, the development of a bespoke prediction modeling is needed that can screen this high depression risk group early, including vulnerable groups such as older adults with low income, based on the results of this study.

Another important finding was that self-recognized stress and subjective health status were independent risk factors for depression in old age. As a person gets old, he or she experiences psychological pressure while going through difficult changes such as retirement from work, separation from children, the onset of various chronic diseases accompanied by physical weakness, and a sense of loss due to the death of close people (e.g., spouse, family, and friends) [35]. When the elderly eventually cannot stand the level of psychological pressure, they feel stressed. It has been reported that stress-related hormones reduce the number of neurotransmitters decreasing neurogenesis in the dentate nucleus of the hippocampus, which results in depression [36]. In particular, older adults tend to first complain of stress symptoms, physical symptoms, health anxiety, difficulty in concentration, and memory impairment, rather than directly complaining of depressive symptoms [37]. If these subjective symptoms complained of by older adults in the high depression risk group are neglected, their depressive symptoms may worsen and this can lead to suicide attempts in extreme cases [38]. Therefore, family members or neighbors of older adults need to continuously communicate with them and listen to the stress symptoms and physical symptoms that the older adults complain about in order to prevent depression. Moreover, when older adults complain of stress or health problems, it is necessary to bring them to a primary healthcare institution for screening.

The limitations of this study are as follows. First, it could not identify the detailed severity of depression or types of depression because it analyzed the prevalence of depression among older adults in the community based on the depression screening test mainly used in epidemiological investigations. Future studies are required to classify the types of depression into minor depressive disorder, subsyndromal depression, and various depressive symptoms using a medical diagnosis, and to explore risk factors according to depression type based on the results of this study. Second, since the food intake frequency survey used the 24-h dietary recall method, there was a possibility of a recall bias. Third, geriatric depression can be affected by social networks such as family and friends, but social networks were not considered. Therefore, future studies are needed to identify risk factors for depression, such as social networks and psychological factors. Fourth, Since the nomogram in this study was developed for the Korean elderly, there is a limit to its application to other cultures or countries. Fifth, since this study is a cross-sectional study, the results cannot be interpreted as a causal relationship, even if risk factors for depression are identified. Additional longitudinal studies are required to prove the causality of the risk factors for depression in older adults in the community found in this study.

## 5. Conclusions

The results of this study implied that it would be necessary to continuously monitor complex risk factors such as household income, skipping breakfast, moderate-intensity physical activity, subjective stress, and subjective health status to prevent depression in older adults living in the community. Furthermore, the establishment of customized prevention policies is needed that can identify high-risk groups of geriatric depression early and continuously manage them.

## Figures and Tables

**Table 1:** General characteristics of the subjects.

Characteristics	*n* (%)
Gender	
Male	1750 (43.6)
Female	2261 (56.4)
Binge(intemperance) frequency	
Non-drinkers	1265 (57.4)
Once a month or fewer	520 (23.6)
Once a week or fewer	265 (12.0)
Almost every day	154 (7.0)
Smoking experience	
Non-smokers	2445 (61.3)
Current smokers	424 (10.6)
Ex-smoker	1122 (28.1)
Living with a spouse	
Yes	2842 (71.5)
No	1132 (28.5)
Education level	
Elementary school graduation or below	2036 (50.8)
Middle school graduation	698 (17.4)
High school graduation	793 (19.8)
College graduation or above	479 (12.0)
Monthly mean household income	
<KRW 1.5 million	1710 (42.8)
≥KRW 1.5 million and <KRW 2 million	376 (9.4)
≥KRW 2 million and <KRW 3 million	600 (15.0)
≥KRW 3 million	1305 (32.7)
Obesity by body mass index (BMI, kg/m^2^)	
Underweight (<18.5 kg/m^2^)	91 (2.3)
Normal (≥18.5 kg/m^2^ and <23 kg/m^2^)	1839 (46.3)
Pre-obesity stage (≥23 kg/m^2^ and <25 kg/m^2^)	1272 (32.0)
Stage 1 obesity (≥25 kg/m^2^ and <30 kg/m^2^)	676 (17.0)
Stage 2 obesity (≥30 kg/m^2^ and <35 kg/m^2^)	92 (2.3)
Stage 3 obesity (≥35 kg/m^2^)	1 (0.1)
Moderate-intensity physical activity	
Yes	146 (3.6)
No	3864 (96.4)
Hypertension	
Normal	777 (19.4)
Prehypertension	871 (21.8)
Hypertension	2352 (58.8)
Diabetes	
Normal	1675 (45.2)
Impaired fasting glucose	1126 (30.4)
Diabetes	901 (24.3)
Hypercholesterolemia	
No	2410 (65.1)
Yes	1292 (34.9)
Hypertriglyceridemia	
No	2720 (84.5)
Yes	498 (15.5)
Waist circumference (cm)	85.3 ± 8.9
Daily n-3 fatty acid intake (g/day)	1.7 ± 1.9
Daily n-6 fatty acid intake (g/day)	6.9 ± 6.0
Daily vitamin c intake (mg/day)	57.7 ± 61.8
Usual hours of sitting per day	10.7 ± 16.1
Usual minutes of sleep per day	444.9 ± 409.7
Major depressive disorder (PHQ-9)	
No	3738 (93.2)
Yes	273 (6.8)

**Table 2:** Characteristics of subjects according to the prevalence of depression, n (%).

Variable	Major Depressive Disorder		*p*
	No (*n* = 3738)	Yes (*n* = 237)	
Age			0.198
60–64	1016 (94.7)	57 (5.3)	
65–69	886 (92.8)	69 (7.2)	
70–74	766 (93.2)	56 (6.8)	
75–79	631 (92.3)	53 (7.7)	
80+	439 (92.0)	38 (8.0)	
Gender			<0.001
Male	1674 (95.7)	76 (4.3)	
Female	2064 (91.3)	197 (8.7)	
Binge(intemperance) frequency			0.540
Non-drinkers	1191 (94.2)	74 (5.8)	
Once a month or fewer	497 (95.6)	23 (4.4)	
Once a week or fewer	248 (93.6)	17 (6.4)	
Almost every day	147 (95.5)	7 (4.5)	
Smoking experience			0.001
Non-smokers	2271 (92.9)	174 (7.1)	
Current smokers	382 (90.1)	42 (9.9)	
Ex-smoker	1067 (95.1)	55 (4.9)	
Living with a spouse			<0.001
Yes	2702 (95.1)	140 (4.9)	
No	1007 (89.0)	125 (11.0)	
Education level			<0.001
Elementary school graduation or below	1846 (90.7)	190 (9.3)	
Middle school graduation	656 (94.0)	42 (6.0)	
High school graduation	762 (96.1)	31 (3.9)	
College graduation or above	470 (98.1)	9 (1.9)	
Monthly mean household income			<0.001
<KRW 1.5 million	1516 (88.7)	194 (11.3)	
≥KRW 1.5 million and <KRW 2 million	353 (93.9)	23 (6.1)	
≥KRW 2 million and <KRW 3 million	579 (96.5)	21 (3.5)	
≥KRW 3 million	1271 (94.7)	34 (2.6)	
Whether or not to receive national basic livelihood security			<0.001
No	3445 (94.1)	215 (5.9)	
Yes	292 (83.4)	58 (16.6)	
Obesity by body mass index (BMI, kg/m^2^)			0.109
Underweight (<18.5 kg/m^2^)	82 (90.1)	9 (9.9)	
Normal (≥18.5 kg/m^2^ and <23 kg/m^2^)	1699 (92.4)	140 (7.6)	
Pre-obesity stage (≥23 kg/m^2^ and <25 kg/m^2^)	1184 (93.1)	88 (6.9)	
Stage 1 obesity (≥25 kg/m^2^ and <30 kg/m^2^)	644 (95.3)	32 (4.7)	
Stage 2 or 3 obesity (≥30 kg/m^2^)	93 (95.7)	4 (4.3)	
Subjective body type perception			<0.001
Very thin	193 (85.0)	34 (15.0)	
Slightly skinny	465 (91.2)	45 (8.8)	
Average	1646 (95.3)	81 (4.7)	
Slightly obese	1155 (93.9)	75 (6.1)	
Very obese	264 (88.0)	36 (12.0)	
Mean frequency of having breakfast per week for the past year			<0.001
5–7 times per week	3091 (94.1)	193 (5.9)	
3–4 times per week	95 (86.4)	15 (13.6)	
1–2 times per week	62 (92.5)	5 (7.5)	
Rarely	99 (82.5)	21 (17.5)	
Control weight over the past year			<0.001
Try to lose weight	1087 (94.4)	64 (5.6)	
Try to maintain weight	691 (95.7)	31 (4.3)	
Try to gain weight	218 (87.2)	32 (12.8)	
Never tried to control weight	1729 (92.3)	144 (7.7)	
Moderate-intensity physical activity			<0.001
Yes	121 (82.9)	25 (17.1)	
No	3616 (93.6)	248 (6.4)	
Days of walking at least 30 min per week			<0.001
Never	904 (88.8)	114 (11.2)	
1 days	217 (93.1)	16 (6.9)	
2 days	325 (96.2)	13 (3.8)	
3 days	415 (92.8)	32 (7.2)	
4 days	256 (94.5)	15 (5.5)	
5 days	329 (95.6)	15 (4.4)	
6 days	170 (97.7)	4 (2.3)	
7 days	1107 (94.7)	62 (5.3)	
Usual level of stress awareness			<0.001
I feel stressed very much	111 (66.9)	55 (33.1)	
I feel stressed a lot	469 (81.1)	109 (18.9)	
I feel stressed a little	1970 (95.9)	85 (4.1)	
I hardly feel stressed	1170 (98.2)	22 (1.8)	
Subjective health status			<0.001
Good	899 (99.1)	8 (0.9)	
Okay	1885 (96.8)	63 (3.2)	
Bad	953 (82.5)	202 (17.5)	
Hypertension			0.502
Normal	731 (94.1)	46 (5.9)	
Prehypertension	812 (93.2)	59 (6.8)	
Hypertension	2184 (92.9)	168 (7.1)	
Diabetes			<0.001
Normal	1583 (94.5)	92 (5.5)	
Impaired fasting glucose	1066 (94.7)	60 (5.3)	
Diabetes	811 (90.0)	90 (10.0)	
Hypercholesterolemia			0.014
No	2270 (94.2)	140 (5.8)	
Yes	1190 (92.1)	102 (7.9)	
Hypertriglyceridemia			0.001
No	2558 (94.0)	162 (6.0)	
Yes	448 (90.0)	50 (10.0)	
Waist circumference (cm)	85.4 ± 8.9	84.6 ± 9.0	0.154
Daily n-3 fatty acid intake (g/day)	1.7 ± 1.8	1.3 ± 2.0	0.001
Daily n-6 fatty acid intake (g/day)	7.0 ± 5.9	5.6 ± 6.2	<0.001
Daily vitamin c intake (mg/day)	58.7 ± 62.6	43.2 ± 48.0	<0.001
Usual hours of sitting per day	10.5 ± 15.9	13.3 ± 18.0	0.007
Usual minutes of sleep per day	444.1 ± 393.1	455.9 ± 592.6	0.647

**Table 3:** The final model for predicting geriatric depression in the community: odds ratio (OR) and 95% confidence interval (CI).

Variables	Unadjusted Model	*p*	Adjusted Model ^1^	*p*
Monthly mean household income				
<KRW 1.5 million (ref)	1.00		1.00	
≥KRW 1.5 million and <KRW 2 million	0.50 (0.32, 0.79)	0.003	0.68 (0.40, 1.14)	0.146
≥KRW 2 million and <KRW 3 million	0.28 (0.17, 0.44)	<0.001	0.30 (0.17, 0.53)	<0.001
≥KRW 3 million	0.20 (0.14, 0.30)	<0.001	0.28 (0.18, 0.43)	<0.001
Mean frequency of having breakfast per week for the past year				
5–7 times per week (ref)	1.00		1.00	
3–4 times per week	2.52 (1.43, 4.44)	0.001	1.87 (0.94, 3.71)	0.071
1–2 times per week	1.29 (0.51, 3.25)	0.587	0.92 (0.32, 2.63)	0.878
Rarely	3.39 (2.07, 5.56)	<0.001	2.14 (1.16, 3.95)	0.015
Moderate-intensity physical activity				
Yes (ref)	1.00		1.00	
No	3.01 (1.92, 4.72)	<0.001	2.05 (1.15, 3.64)	0.014
Usual level of stress awareness				
I hardly feel stressed (ref)	1.00		1.00	
I feel stressed very much	26.35 (15.48, 44.83)	<0.001	14.17 (7.71, 26.02)	<0.001
I feel stressed a lot	12.36 (7.72, 19.78)	<0.001	8.00 (4.75, 13.46)	<0.001
I feel stressed a little	2.29 (1.42, 3.68)	0.001	2.18 (1.30, 3.66)	0.003
Subjective health status				
Good (ref)	1.00		1.00	
Okay	3.75 (1.79, 7.87)	<0.001	2.65 (1.18, 5.95)	0.018
Bad	23.81 (11.68, 48.56)	<0.001	11.21 (5.14, 24.40)	<0.001

^1^ Adjusted for monthly mean household income, mean frequency of having breakfast per week for the past year, moderate-intensity physical activity, usual level of stress awareness, and subjective health status.

## Data Availability

Restrictions apply to the availability of these data. Data was obtained from National Health and Nutrition Examination Survey and are available [from the National Health and Nutrition Examination Survey/https://data.go.kr/en/data/15076556/fileData.do (accessed on 1 July 2021) with the permission of Korea Centers for Disease Control and Prevention.
